# miR‐21 promotes cardiac fibroblast‐to‐myofibroblast transformation and myocardial fibrosis by targeting Jagged1

**DOI:** 10.1111/jcmm.13654

**Published:** 2018-05-28

**Authors:** Xue‐liang Zhou, Hua Xu, Zhi‐bo Liu, Qi‐cai Wu, Rong‐rong Zhu, Ji‐chun Liu

**Affiliations:** ^1^ Department of Cardiac Surgery The First Affiliated Hospital Nanchang University Nanchang China; ^2^ Department of Obstetrics and Gynecology Jiangxi Province hospital of integrated traditional Nanchang 330006 China

**Keywords:** Jagged1, miR‐21, myocardial fibrosis, TGF‐β1

## Abstract

Myocardial fibrosis after myocardial infarction (MI) is a leading cause of heart diseases. MI activates cardiac fibroblasts (CFs) and promotes CF to myofibroblast transformation (CMT). This study aimed to investigate the role of miR‐21 in the regulation of CMT and myocardial fibrosis. Primary rat CFs were isolated from young SD rats and treated with TGF‐β1, miR‐21 sponge or Jagged1 siRNA. Cell proliferation, invasion and adhesion were detected. MI model was established in male SD rats using LAD ligation method and infected with recombinant adenovirus. The heart function and morphology was evaluated by ultrasonic and histological analysis. We found that TGF‐β1 induced the up‐regulation of miR‐21 and down‐regulation of Jagged1 in rat CFs. Luciferase assay showed that miR‐21 targeted 3′‐UTR of Jagged1 in rat CFs. miR‐21 sponge inhibited the transformation of rat CFs into myofibroblasts, and abolished the inhibition of Jagged1 mRNA and protein expression by TGF‐β1. Furthermore, these effects of miR‐21 sponge on rat CFS were reversed by siRNA mediated knockdown of Jagged1. In vivo, heart dysfunction and myocardial fibrosis in MI model rats were partly improved by miR‐21 sponge but were aggravated by Jagged1 knockdown. Taken together, these results suggest that miR‐21 promotes cardiac fibroblast‐to‐myofibroblast transformation and myocardial fibrosis by targeting Jagged1. miR‐21 and Jagged1 are potential therapeutic targets for myocardial fibrosis.

## 1 INTRODUCTION

Myocardial infarction (MI) is a leading cause of sudden death. Myocardial fibrosis is an important pathological change after MI, and excess fibrosis could lead to ventricular dilation, infarct expansion, and heart failure and even death.^1^, ^2^ Therefore, inhibiting or limiting cardiac fibrosis after MI is important to imporving heart fucntion and reducing the mortality.^3^, ^4^

MicroRNAs (miRNAs) are 20‐23 nucleotides long and act as negative regulator of gene expression by enhancing mRNA degradation or/and inhibiting mRNA translation.^5^ Due to their power to modulate gene expression, it is not surprising that miRNAs participate in various pathological processes, including myocardial fibrosis.^6^, ^7^, ^8^ Among several miRNAs implicated in myocardial fibrosis, miR‐21 has attracted particular attention because its targets play important role in the fibrosis in multiple organs such as the kidney, the liver and the lung.^9^, ^10^, ^11^ A very recent study reported that miR‐21 promoted cardiac fibrosis after MI via targeting Smad7.^12^ This finding is expected given that transforming growth factor‐β1 (TGF‐β1) signalling is a key regulator of cardiac repair and fibrosis after MI. TGF‐β1 is significantly up‐regulated in cardiac fibroblasts (CFs) after MI and it promotes CF to myofibroblast transformation (CMT) through TGF‐β1/Smad3 pathway. During CMT, CFs are activated to transform into myofibroblasts that highly express α‐smooth muscle actin (α‐SMA), leading to excessive collagen deposition in fibrotic myocardium.^13^, ^14^

Considering that miRNAs usually target a variety of targets to regulate physiological and pathological processes, we wondered that miR‐21 may target other signalling pathways involved in myocardial fibrosis. Notch is a highly conserved intercellular signalling pathway and has been implicated in tissue fibrosis in various disease conditions such as scleroderma, lung fibrosis, kidney fibrosis, liver fibrosis and cardiac fibrosis.^15^, ^16^ Interestingly, Notch ligand jagged1 has been suggested as a direct target of miR‐21 in breast cancer cells.^17^ However, the direct link between miR‐21 and Notch signalling in the regulation of myocardial fibrosis remains unclear. Therefore, in this study we aimed to investigate whether miR‐21 regulates myocardial fibrosis by targeting Notch/Jagged1 signalling pathway.

## 2 MATERIALS AND METHODS

### 2.1 Culture of primary rat CFs

All animal experiments were performed in accordance with the guidelines of NIH and under approved protocols of the Animal Care and Use Committee of Nanchang University. Primary rat CFs were isolated from Sprague‐Dawley rats (1‐3 days old) using a protocol previously described.^18^ CFs were cultured in Dulbecco's modified Eagle's medium (DMEM, HyClone) supplemented with 10% foetal bovine serum (FBS) in a humidified atmosphere with 5% CO2 at 37°C. Only CFs with no more than three passages were used in this study.

### 2.2 Construction of recombinant adenoviruses

Recombinant adenoviruses expressing miR‐21 (NR_031823.1), miR‐21 sponge (5′ TCAACATCAGTCTGATAAGCTATCAACATCAGTCTGATAAGCTATCAACATCAGTCTGATAAGCTA 3′) and Jagged1 shRNA (5′ GGGATTCCAGTAATGACACTATTCA 3′) were prepared by Hanbio Biotechlogy Co., Ltd. (Hanbio, Shanghai, China) using pAdEasyTM vector system (Qbiogene, US). Briefly, cDNA or sponge sequences of miR‐21 were subcloned into a shuttle vector pTrack‐CMV (Qbiogene, US), Jagged1 shRNA was subcloned into a shuttle vector pTrack‐U6 (Qbiogene, US). The pAdTrack‐CMV‐miR‐21, pAdTrack‐CMV‐miR‐21 sponge, pAdTrack‐U6‐shJagged1 and pAdEasy‐1 (Qbiogene, US) expressing GFP were homologously recombined in bacteria BJ5183 and the recombinant plasmids were transfected into HEK293 cells by using LipofiterTM transfection reagent (Hanbio, Shanghai, China) to generate recombinant adenoviruses. Empty adenoviruses (Ad‐GFP and siNC) was used as a control. Ad‐miR‐21, Ad‐miR‐21 sponge, Ad‐shJag1 (siJag1) and Ad‐GFP were propagated in HEK293 cells, purified and the titre of virus was measured by plaque assays. For adenovirus infection, the MOI was 50 for all adenovirus and the medium was replaced 4 hours post‐infection with complete medium.

### 2.3 Rat MI model

Myocardial infarction model was established in male Sprague‐Dawley (SD) rats using left anterior descending coronary artery (LAD) ligation method as described previously.^19^ The rats were anesthetized with sodium pentobarbital (60 mg/kg, ip) and a thoracotomy was performed. A 26‐gauge needle containing 200 μL of diluted adenoviruses (3 × 10^10^ pfu/mL) or sterile saline was advanced from the apex of the left ventricle to the aortic root. The aorta and main pulmonary arteries were clamped for 10 seconds distal to the site of the injector and solution injected, and then the chest was closed. The hearts underwent hemodynamic studies at day 4 after the injection of the adenovirus.

### 2.4 Echocardiographic measurement

At the time points of before surgery, 4 weeks after the surgery, echocardiography was conducted. Rats were anaesthetized with an ip injection of pentobarbital sodium (40 mg/kg) and fixed in supine position on a heating pad. Heart rate was monitored with a standard limb lead II electrocardiogram (ECG) and maintained at 50 ± 5 per minute during the echocardiography. Cardiac functions were evaluated by transthoracic echocardiography with an ultrasound machine (Visual Sonics Inc., Toronto, Canada) with a 716 probe. Left ventricular systolic diameter (ESD), Left ventricular diastolic diameter (EVD), ejection fraction (EF) and fractional shortening (FS) were derived automatically by the High‐Resolution Electrocardiograph System. All measurements were averaged over three consecutive cardiac cycles.

### 2.5 Histological analysis

After rats were adequately anesthetized, thoracotomy was conducted and the hearts were collected. For histological staining, the hearts were sliced along the short‐axis plane at the level of one‐third of the distance from the atrioventricular ring to the apex. The hearts were then fixed in 4% paraformaldehyde, embedded with paraffin, and cut into 5 mm thick sections along the centre of the fibrotic scar. Masson's trichrome staining was performed to evaluate collagen deposition. Five sections were randomly selected from each rat of total three rats in each group. All quantitative evaluations were carried out by ImagePro Plus software (version 6.0, Media Cybernetics, Bethesda, MD, USA).

### 2.6 CCK‐8 assay

CFs were seeded in 96‐well culture plate at a density of 2 × 10^3^ cells/well and cultured in a humidified chamber at 37°C. Each day for seven consecutive days, viable cells were detected with CCK‐8 Assay (Dojindo, Japan) according to the manufacturer's instructions. CCK‐8 solution was added to the cells in 96‐well plate and the plates were incubated at 37°C for 4 hours, and the optical density of each well was read at 450 nm using a microplate reader (Molecular Devices, USA).

### 2.7 Cell invasion assay

Cardiac fibroblasts were resuspended in DMEM supplemented with 1% FBS at density of 5 × 10^5^/mL. 100 μL of cell suspension was added to the upper chamber of Transwell (Corning) while DMEM supplemented with 20% FBS was added to the lower chamber. After incubation for 48 hours, the cells on the bottom of chamber membrane were fixed in methanol for 30 minutes, stained with 0.1% crystal violet (Sigma) for 20 minutes, and counted under a microscope.

### 2.8 PCR

Total RNA was extracted from CFs using Trizol (Invitrogen). RNA concentration and purity was determined using Thermo NanoDrop 2000. cDNAs were synthesized using 1 μg total RNA, reverse transcriptase (Fermentas) and Oligo‐dT primer. Real‐time PCR was performed using SYBR Premix (Invitrogen) and following primers: Vimentin 5′ ACTAATGAGTCCCTGGAGCG 3′ and 5′ AGGTGGCGATCTCAATGTCA 3′; DDR2 5′ GCCAACAAGAATGCCAGGAA 3′ and 5′ CGTAACTGACTGTGGCATT 3′; α‐SMA 5′ GCTATTCAGGCTGTGCTGTC 3′ and 5′ GGTAGTCGGTGAGATCTCGG 3′, Tensin 5′ CACACCAGCCTTACCAGAGA 3′ and 5′ AAGTCTGGAAGCGTGTGAGA 3′, GAPDH 5′ AGTCTACTGGCGTCTTCACC 3′ and 5′ CCACGATGCCAAAGTTGTCA 3′, Jagged1 5′ GGCTACTCAGGACCGAACTG 3′ and 5′GCACACGCACTTGAATCCAT 3′ miR‐21 5′ GGGGGTACCCTTCAGGAAGCTGGTTTC3′ and 5′ GGGGATATCTACATGTGAGGCAGGTTCTCAC 3′, U6 5′ CGCTTCGGCACATATACTA 3′ and 5′ CGCTTCACGAATTTGCGTGTCA 3′. PCR was run on Applied Biosystems 7900 QPCR system with the cycling parameters: 95°C 5 minutes 1 cycle; 95°C 10 seconds, 60°C 20 seconds, 40 cycles.

### 2.9 Western blot analysis

Total protein was isolated from CFs and quantitated by BSA method. 50 μg proteins were separated on a 10% SDS‐PAGE and transferred to PVDF membranes (Millipore, USA). The membranes were incubated with antibodies for Vimentin, DDR2, α‐SMA, tensin and GAPDH (all from CST) and antibodies for Jagged1 (from Abcam) at 4°C overnight. The membranes were washed with TBST and then incubated with HRP conjugated secondary antibodies (Sigma, USA) at room temperature for 1 hour. The membranes were exposed using ECL kit (Pierce, Rockford, IL, USA) and blots were quantified using Image.plus5.1 software.

### 2.10 3′‐UTR luciferase assay

A 1 300‐bp fragment of Jagged1 3′‐UTR, which contains the putative binding site for miR‐21, and corresponding fragment containing mutant binding site were inserted into pGL3 vector to make pGL3‐Jagged1‐WT‐3′‐UTR and pGL3‐ Jagged1‐3′‐UTR‐Mut constructs. The constructs were confirmed by DNA sequence analysis. CFs were plated (8 × 10^4^ cells/well) in 24‐well plate and cultured overnight, then pGL3‐ Jagged1‐3′‐UTR or pGL3‐ Jagged1‐3′‐UTRmut construct (20 ng) was cotransfected with 2 ng Renilla luciferase vector, pRL‐TK (Promega), and 200 ng of miR‐21 overexpression vector or control vector. Cells were lysed 48 hours after transfection and luciferase activities were analysed using Dual‐Luciferase Reporter Assay System (Promega) following the manufacturer's instructions.

### 2.11 Statistical analysis

All data were presented as mean ± SD and analysed by SPSS 11.0 statistical software. Differences among various treatment groups were compared by one‐way ANOVA.

Differences were considered significant at *P* < .05.

## 3 RESULTS

### 3.1 miR‐21 is up‐regulated by TGF‐β1 and targets Jagged1 in rat CFs

**Figure 1:**
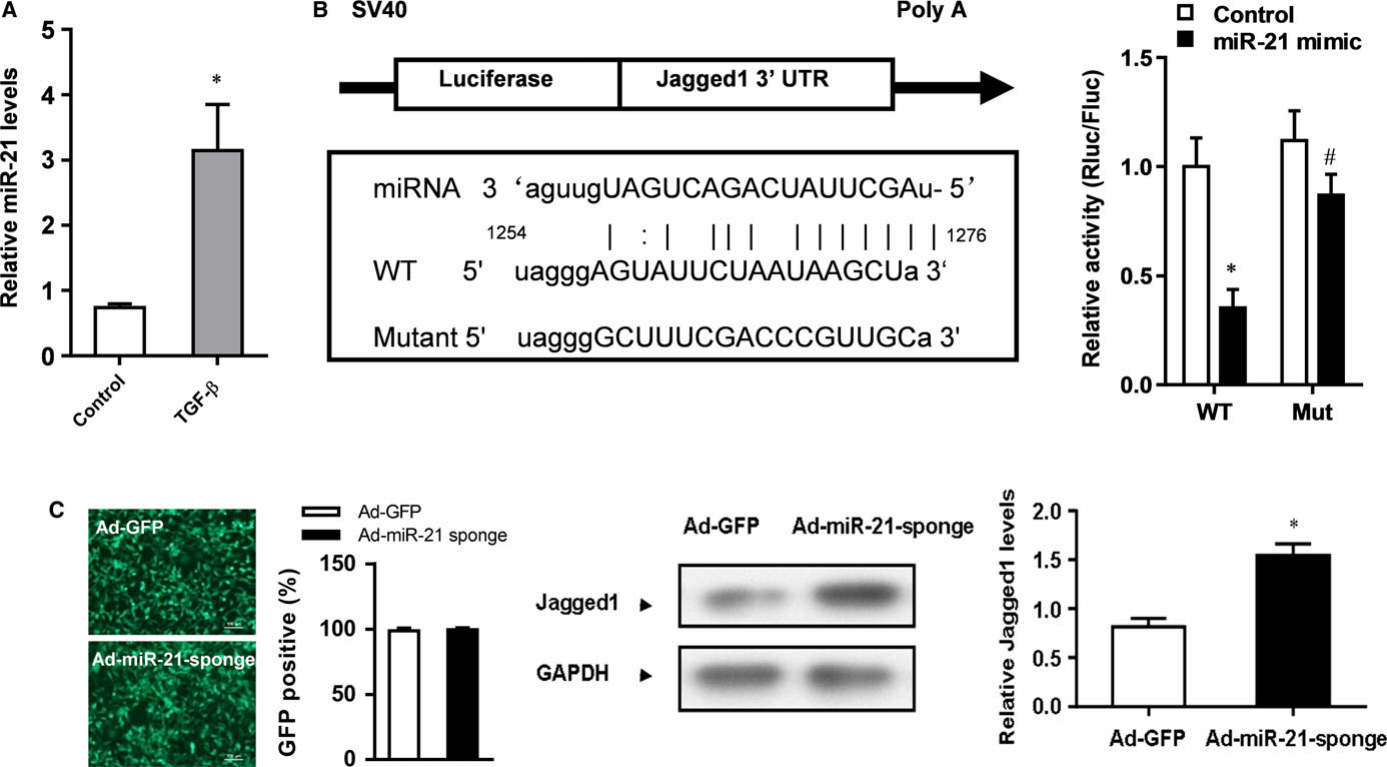
miR‐21 is up‐regulated by TGF‐β1 and targets Jagged1 in rat CFs. A, Rat CFs were treated with TGF‐β1 and the expression of miR‐21 was detected by real‐time PCR. *P < .05 vs Control. B, Left: Jagged1 3′‐UTR luciferase reporter construct. Right: Luciferase assay of luciferase activity in CFs treated with control or miR‐21 mimic. Data were presented as mean ± SD (n = 3). *P < .05 vs Control in WT, ^#^ *P* > .05 vs Control in Mut. C, Rat CFs were treated with Ad‐GFP as control or Ad‐miR‐21 sponge, and the efficiency of adenovirus transduction was measured based on GFP positive cells. The protein expression of Jagged1 was detected by Western blot analysis. GAPDH was loading control. Data were presented as mean ± SD (n = 3). *P < .05 vs Ad‐GFP

To understand the role of miR‐21 in CMT, first we treated rat CFs with TGF‐β1 which is known to induce CMT. We observed that TGF‐β1 significantly increased the expression of miR‐21 (Figure 1A). Because miR‐21 has been shown to target Notch ligand jagged1 in breast cancer cells,^17^ we wondered whether miR‐21 also targets jagged1 in CFs. We predicted putative binding site of miR‐21 in 3′‐UTR of Jagged1 and made wild‐type and mutant luciferase reporter constructs. Luciferase assay showed that miR‐21 mimic significantly inhibited luciferase activity of wild‐type Jagged1 3′‐UTR construct but had no significant effects on the activity of mutant Jagged1 3′‐UTR construct in CFs (Figure 1B). Furthermore, Jagged1 protein level was significantly higher in rat CFs treated with adenovirus that carried miR‐21 sponge (inhibitor) than in rat CFs treated with adenovirus that carried GFP as the control (Figure 1C). Collectively, these data suggest that TGF‐β1 induces the expression of miR‐21 which then inhibits the expression of Jagged1.

### 3.2 miR‐21 promotes CMT of rat CFs

**Figure 2:**
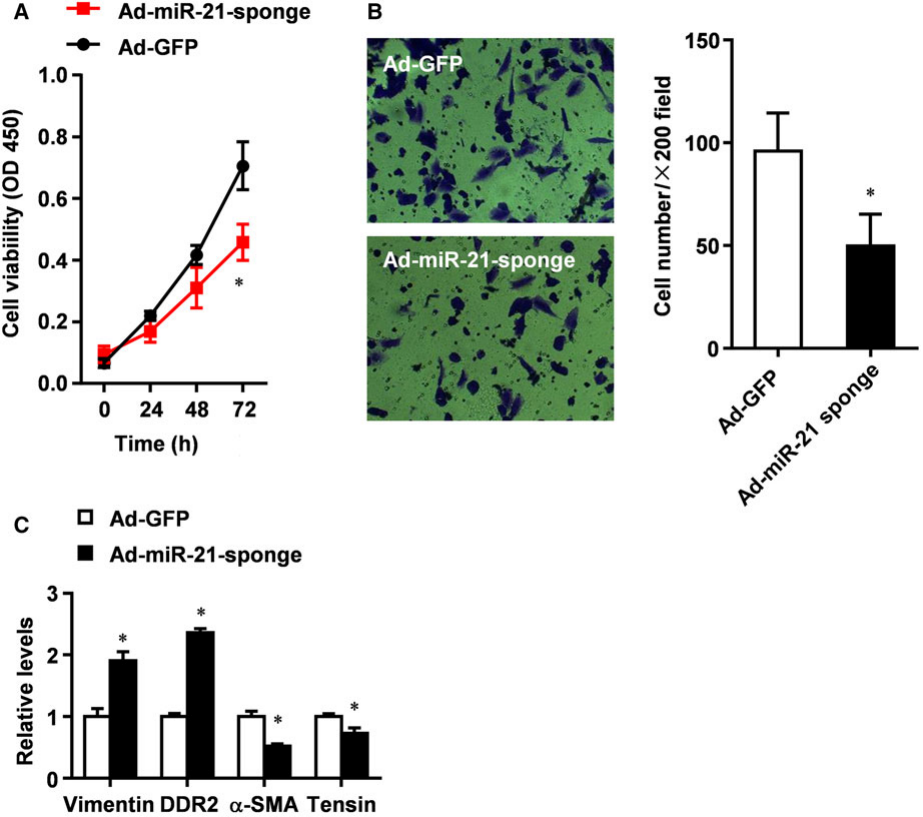
miR‐21 promotes CMT of rat CFs. A**,** CCK8 assay of the proliferation of CFs treated with Ad‐GFP as control or Ad‐miR‐21 sponge. B, Transwell assay of the invasion of CFs treated with Ad‐GFP as control or Ad‐miR‐21 sponge. C, Real‐time PCR analysis of the expression of CMT markers in CFs treated with Ad‐GFP as control or Ad‐miR‐21 sponge. All data were presented as mean ± SD (n = 3). *P < .05 vs Ad‐GFP

Next we examined the changes of the phenotypes of rat CFs after the inhibition of miR‐21. We found that Ad‐miR‐21 sponge significantly reduced the proliferation of CFs (Figure 2A). Furthermore, Transwell assay showed that Ad‐miR‐21 sponge significantly decreased the invasion of CFs (Figure 2B). In addition, real‐time PCR analysis showed that Ad‐miR‐21 sponge significantly increased the expression of fibroblast markers vimentin and Discoidin Domain Receptor 2 (DDR2) but decreased the expression of myofibroblast markers α‐SMA and tensin (Figure 2C). These data indicate that miR‐21 promotes the transition of rat CFs into myofibroblasts.

### 3.3 miR‐21 mediates the effects of TGF‐β1 to promote the proliferation and invasion and inhibit Jagged1 expression in CFs

**Figure 3:**
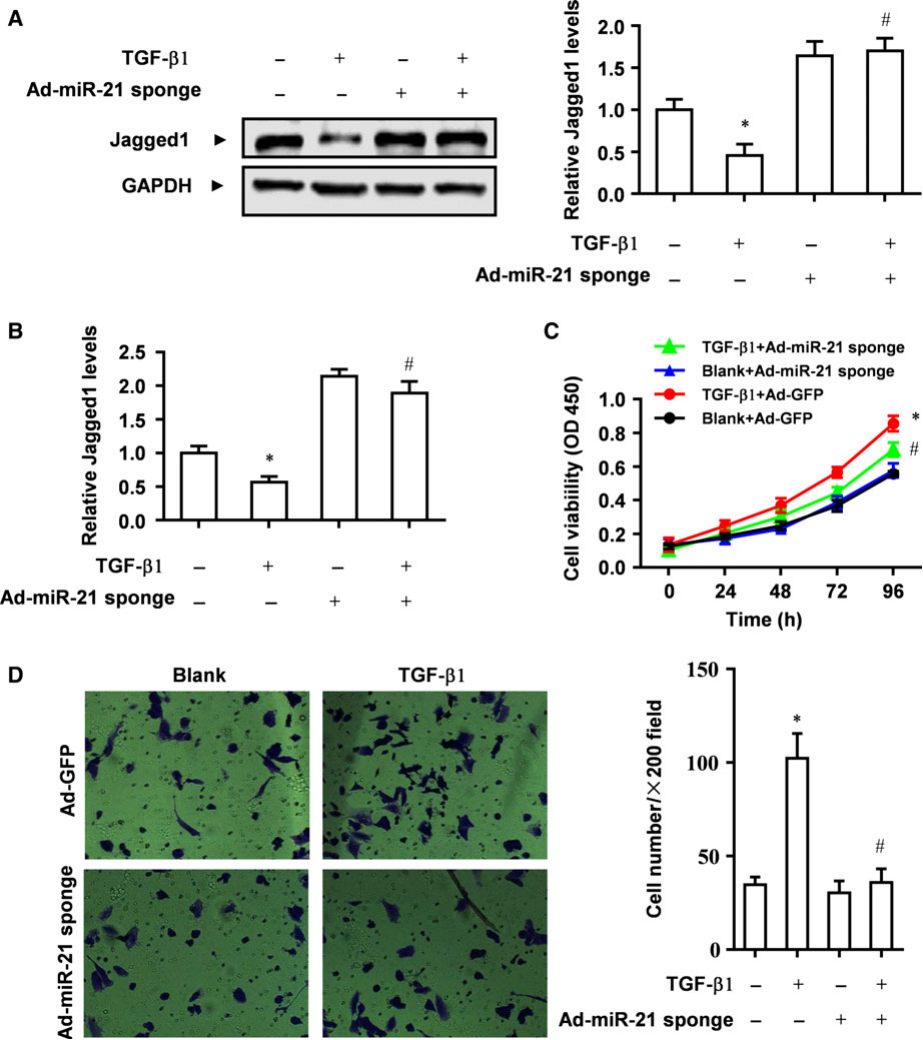
miR‐21 inhibitor abrogates the biological effects of TGF‐β1 in rat CFs. A, Rat CFs were treated with TGF‐β1 or/and Ad‐miR‐21 sponge, and protein expression of Jagged1 was detected by Western blot analysis. GAPDH was loading control. Data were presented as mean ± SD (n = 3). *P < .05 vs Control without any treatment. ^#^ *P* > .05 vs Ad‐miR‐21 sponge only. B, mRNA expression of Jagged1 was detected by real‐time PCR. Data were presented as mean ± SD (n = 3). *P < .05 vs Control without any treatment. ^#^ *P* > .05 vs Ad‐miR‐21 sponge only. C**,** CCK8 assay of the proliferation of CFs treated as indicated. *P < .05 vs Blank+ Ad‐GFP. ^#^ *P* > .05 vs Blank +Ad‐miR‐21 sponge. D, Transwell assay of the invasion of CFs treated as indicated. Data were presented as mean ± SD (n = 3). *P < .05 vs Blank+Ad‐GFP, ^#^ *P* > .05 vs Blank+Ad‐miR‐21 sponge

Since TGF‐β1 induced miR‐21 expression, we wondered whether biological effects of TGF‐β1 on CFs are mediated by miR‐21. Western blot analysis showed that TGF‐β1 inhibited Jagged1 protein expression in rat CFs, but the inhibition was abolished in CFs treated with Ad‐miR‐21 sponge (Figure 3A). Moreover, real‐time PCR showed that TGF‐β1 inhibited Jagged1 mRNA expression in rat CFs, but the inhibition was abolished in CFs treated with Ad‐miR‐21 sponge (Figure 3B).

While TGF‐β1 treatment stimulated the proliferation of CFs, Ad‐miR‐21 sponge significantly attenuated the stimulating effect of TGF‐β1 on the proliferation of CFs (Figure 3C). By transwell assay we found that TGF‐β1 treatment stimulated the invasion of CFs, but Ad‐miR‐21 sponge nearly abolished the stimulating effect of TGF‐β1 on the invasion of CFs (Figure 3D). Taken together, these results suggest that biological effects of TGF‐β1 on CFs are mediated by miR‐21.

### 3.4 miR‐21 mediates TGF‐β1 induced changes of CMT markers of rat CFs

**Figure 4:**
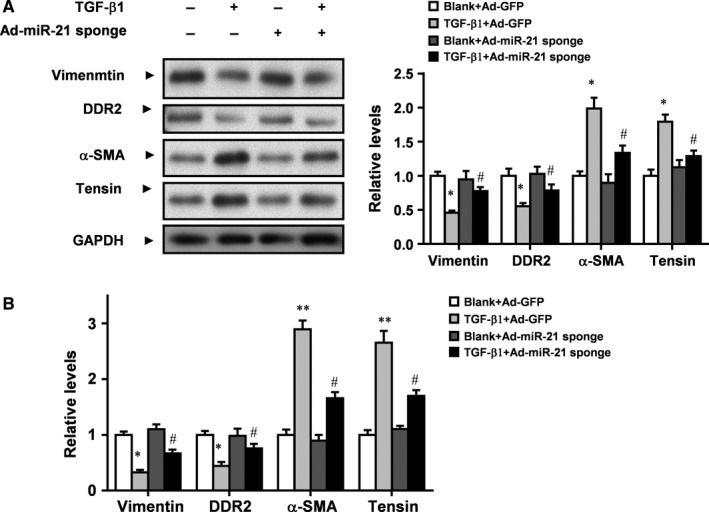
miR‐21 inhibitor abrogates TGF‐β1 induced changes of CMT markers in rat CFs. A, Western blot analysis of the expression of CMT markers in rat CFs treated as indicated. GAPDH was loading control. B, Real‐time PCR analysis of the expression of CMT markers in rat CFs treated as indicated. Data were presented as mean ± SD (n = 3). *P < .05 vs Blank+Ad‐GFP, ^#^ *P* > .05 vs Blank+Ad‐miR‐21 sponge

To understand whether miR‐21 mediates TGF‐β1 induced CMT of rat CFs, we detected the markers of CMT. Western blot analysis showed that TGF‐β1 decreased protein levels of fibroblast markers vimentin and DDR2 but increased protein levels of myofibroblast markers α‐SMA and tensin. However, Ad‐miR‐21 sponge increased protein levels of vimentin and DDR2 but decreased protein levels of α‐SMA and tensin in CFs treated with TGF‐β1 (Figure 4A). Real‐time PCR confirmed that TGF‐β1 decreased mRNA expression of fibroblast markers vimentin and DDR2 but increased mRNA expression of myofibroblast markers α‐SMA and tensin. However, Ad‐miR‐21 sponge increased mRNA expression of vimentin and DDR2 but decreased mRNA expression of α‐SMA and tensin in CFs treated with TGF‐β1 (Figure 4B). Taken together, these data suggest that miR‐21 mediates TGF‐β1 induced CMT of rat CFs.

### 3.5 miR‐21 mediates TGF‐β1 induced CMT by targeting Jagged1

**Figure 5:**
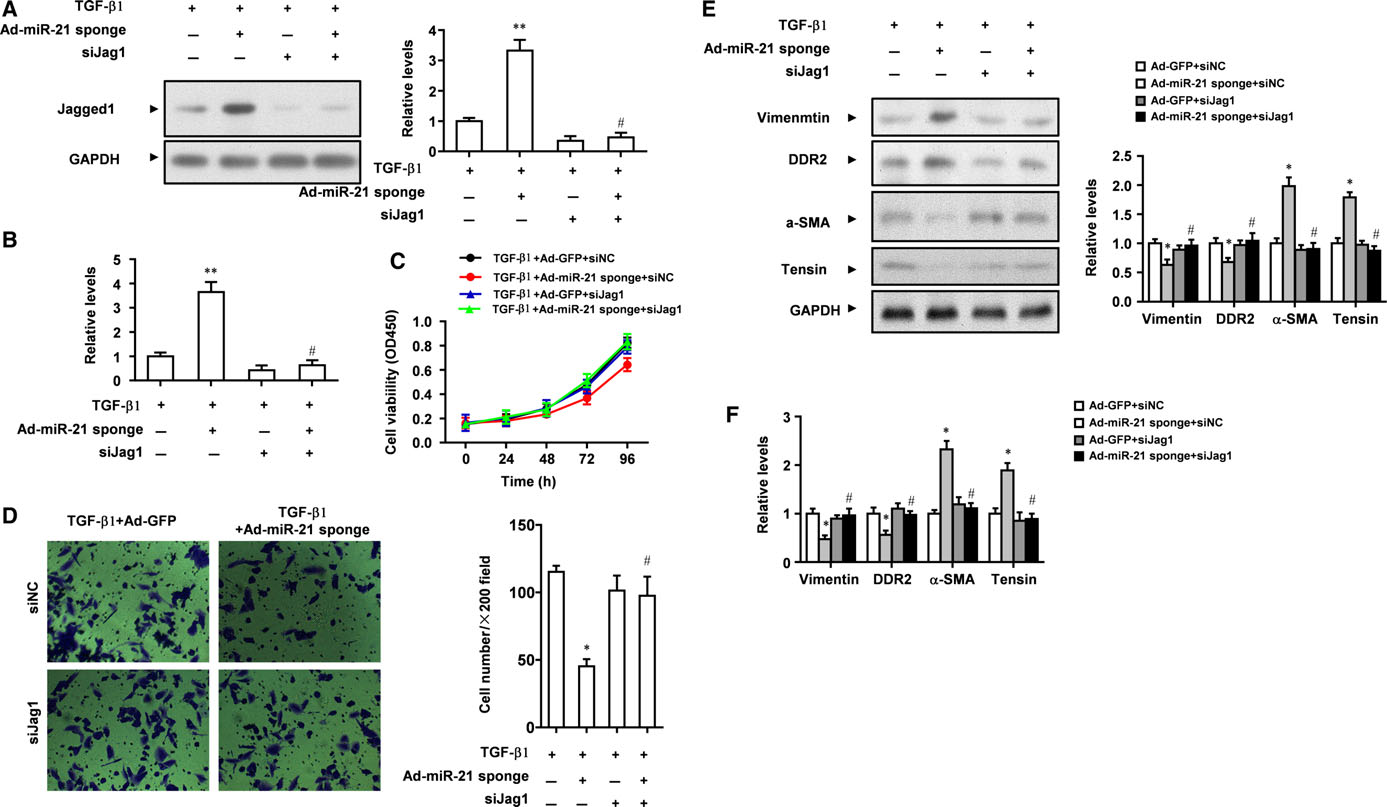
Knockdown of Jagged1 antagonizes the biological effects of miR‐21 inhibitor in rat CFs. A, Rat CFs were treated with TGF‐β1, Ad‐miR‐21 sponge or/and siRNA for Jagged1, and protein expression of Jagged1 was detected by Western blot analysis. GAPDH was loading control. Data were presented as mean ± SD (n = 3). **P < .01 vs TGF‐β1 treatment only. ^#^ *P* > .05 vs TGF‐β1+ siJag1. B, mRNA expression of Jagged1 was detected by real‐time PCR. Data were presented as mean ± SD (n = 3). **P < .01 vs TGF‐β1 treatment only. ^#^ *P* > .05 vs TGF‐β1+ siJag1. C**,** CCK8 assay of the proliferation of CFs treated as indicated. D, Transwell assay of the invasion of CFs treated as indicated. Data were presented as mean ± SD (n = 3). *P < .05 vs TGF‐β1 treatment only. ^#^ *P* > .05 vsTGF‐β1+ siJag1. E, Western blot analysis of the expression of CMT markers in rat CFs treated as indicated. GAPDH was loading control. F, Real‐time PCR analysis of the expression of CMT markers in rat CFs treated as indicated. Data were presented as mean ± SD (n = 3). *P < .05 vs Ad‐GFP+siNC, ^#^ *P* > .05 vs Ad‐GFP+siJag1

To test our hypothesis that miR‐21 mediates TGF‐β1 induced CMT by targeting Jagged1, first we detected the expression of Jagged1 in CFs treated with TGF‐β1, Ad‐miR‐21 sponge, or/and siJagged1. TGF‐β1 inhibited Jagged1 protein expression and this was antagonized by Ad‐miR‐21 sponge, and Jagged1 protein expression was very low after treatment with siJagged1 (Figure 5A). Similarly, TGF‐β1 inhibited Jagged1 mRNA expression and this was antagonized by Ad‐miR‐21 sponge, and Jagged1 mRNA expression was very low after treatment with siJagged1 (Figure 5B).

Next we examined the phenotypes of CFs treated with TGF‐β1, Ad‐miR‐21 sponge, or/and siJagged1. We found that Ad‐miR‐21 sponge significantly reduced TGF‐β1 induced proliferation of CFs, but the inhibition was abolished by siJagged1 (Figure 5C). Transwell assay showed that Ad‐miR‐21 sponge significantly decreased TGF‐β1 induced invasion of CFs, but the inhibition was abolished by siJagged1 (Figure 5D). Furthermore, we detected CMT markers in CFs treated with TGF‐β1, Ad‐miR‐21 sponge, or/and siJagged1. Western blot analysis showed that Ad‐miR‐21 sponge significantly increased protein levels of fibroblast markers vimentin and DDR2 and decreased protein levels of myofibroblast markers α‐SMA and tensin, but these changed were reversed by siJagged1 (Figure 5E). Similarly, Ad‐miR‐21 sponge significantly increased mRNA levels of vimentin and DDR2 and decreased mRNA levels of α‐SMA and tensin, but these changed were reversed by siJagged (Figure 5F). Collectively, these data indicate that TGF‐β1 induced CMT is mediated by miR‐21 through the targeting of Jagged1 by miR‐21.

### 3.6 miR‐21 promotes myocardial fibrosis in vivo by targeting Jagged1

**Figure 6:**
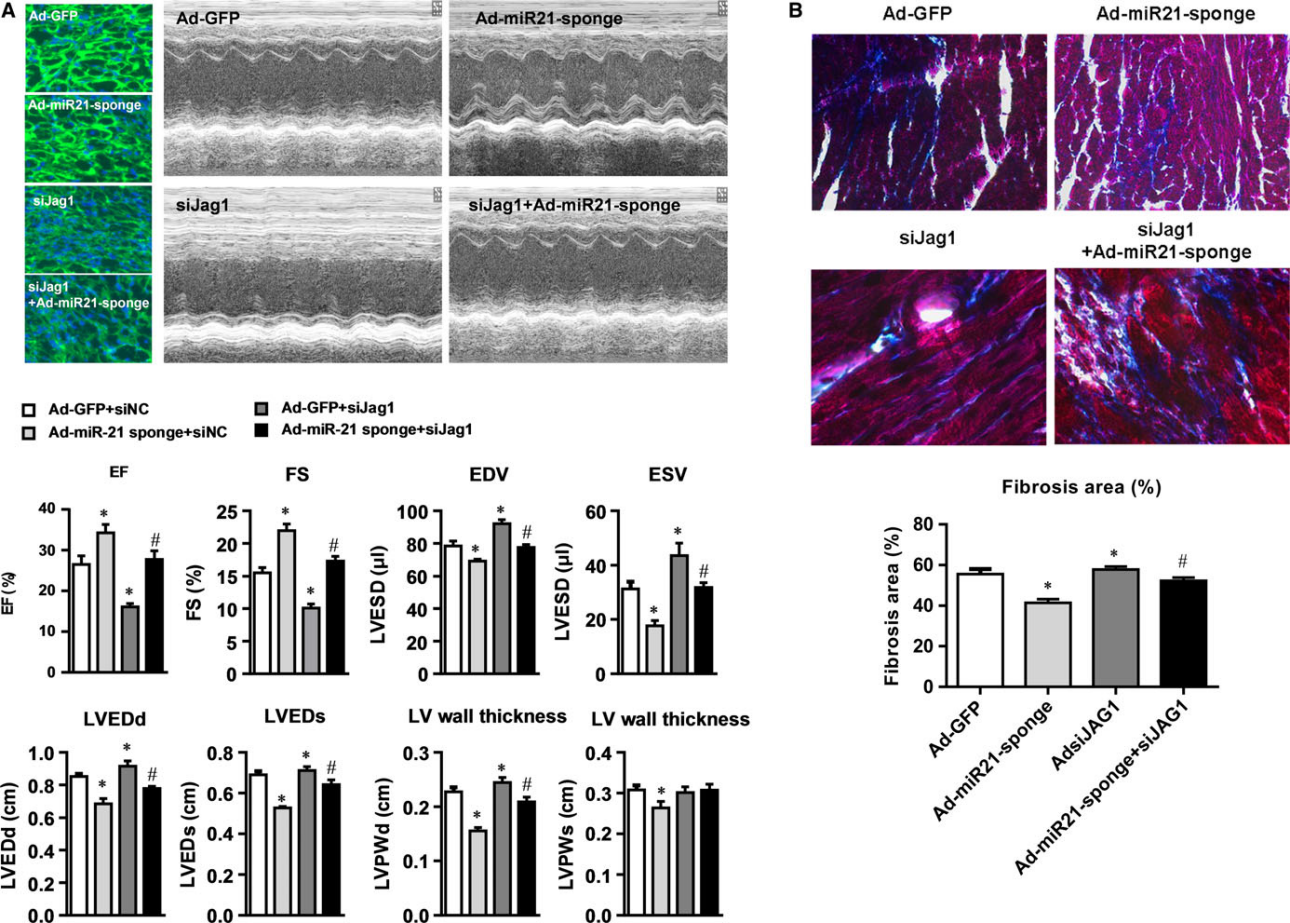
miR‐21 inhibitor improves while Jagged1 knockdown aggravates myocardial fibrosis in rat MI model. A. Measurements of cardiac function indexes of the rats injected with different adenovirus vectors. EF, left ventricular ejection fraction; FS, left ventricular fractional shortening; EDV, left ventricular end‐diastolic volume; ESV, left ventricular end‐systolic volume; LVEDd, left ventricular internal dimensions at end diastole; LVEDs, left ventricular internal dimensions at end systole; LVPWd, left ventricular posterior wall end diastole; LVPWs, left ventricular posterior wall end systole. All data were presented as mean ± SD (n = 3). *P < .05 vs Ad‐GFP, ^#^ *P* > .05 vs Ad‐GFP. B, Top: Representative images of Masson staining of heart tissues of the rats injected with different adenovirus vectors. Amplification: ×400. Bottom: Quantitative analysis of fibrosis area in each group of rats (n = 3)

Finally, we examined the functional interaction of miR‐21 and Jagged1 in vivo in rat MI model. In control MI model rats treated with Ad‐GFP, we observed obvious heart dysfunction. The dysfunction in model rats was partly improved by treatment with Ad‐miR‐21 sponge (knockdown of miR‐21) but was aggravated by Ad‐shJAG1 (knockdown of Jagged1), and was nearly the same as that in rat treated with both Ad‐miR‐21 sponge and Ad‐shJAG1 because knockdown of miR‐21 and knockdown of Jagged1 neutralized each other (Figure 6A). Histological analysis showed myocardial fibrosis in model rats treated with Ad‐GFP, myocardial fibrosis was partly improved by treatment with Ad‐miR‐21 sponge but was aggravated by Ad‐shJAG1, and was nearly the same as that in rat treated with both Ad‐miR‐21 sponge and Ad‐shJAG1 (Figure 6B). Taken together, these results indicate that miR‐21 promotes myocardial fibrosis in vivo by targeting Jagged1.

## 4 DISCUSSION

Myocardial fibrosis is an important pathological process following heart attack. Currently it is generally considered that CMT is a crucial event in the initiation of myocardial fibrosis. TGF‐β1 signalling is a well‐known pathway that could induce CMT and promotes myocardial fibrosis.^4^ On the other hand, recent studies indicate that Notch signalling inhibits CMT and alleviates myocardial fibrosis.^18^, ^19^ However, the underlying mechanism by which TGF‐β1 and Notch signalling pathways crosstalk to regulate CMT remains not fully understood.

Interestingly, emerging evidence suggests the role of miRNAs in the regulation of myocardial fibrosis.^6^, ^7^, ^8^ Therefore, in this study we aimed to characterize the role of miR‐21 in mediating the regulation of CMT by TGF‐β1 and Notch signalling pathways. For the first we identified miR‐21 as a key molecule that links TGF‐β1 and Notch signalling pathways in the regulation of CMT. Our results demonstrated that miR‐21 was induced by TGF‐β1, and it targeted Jagged1 to inhibit its expression and the activation of Notch signalling. Functionally, we found that miR‐21 mediated the effects of TGF‐β1 to promote the proliferation and invasion and CMT in CFs, and these effects were attributed to the targeting of Jagged 1 by miR‐21. Finally, in rat model we confirmed that miR‐21 promoted myocardial fibrosis by targeting Jagged1.

In this study, we first demonstrated that TGF‐β1 induced miR‐21 expression but inhibited Jagged1 expression, next we wondered whether biological effects of TGF‐β1 are mediated by miR‐21. The results showed that Ad‐miR‐21 sponge attenuated the stimulating effects of TGF‐β1 on the proliferation and invasion of CFs, but antagonized the inhibition of Jagged1 expression in CFs by TGF‐β1. Furthermore, Western blot and PCR analysis showed that TGF‐β1 decreased the expression of fibroblast markers vimentin and DDR2 but increased the expression of myofibroblast markers α‐SMA and tensin, and these changes of CMT markers were antagonized by Ad‐miR‐21 sponge. These data indicate that loss of miR‐21 would block TGF‐β1 induced effects on the phenotypes and gene expression in CFs. In other words, miR‐21 mediates TGF‐β1 induced CMT.

We went on to demonstrate that siRNA mediated knockdown of Jagged1 could abolish the effects of Ad‐miR‐21 sponge to up‐regulate Jagged1 expression and inhibit CMT in rat CFs. Furthermore, in rat MI models we demonstrated that heart dysfunction and myocardial fibrosis got aggravated by Ad‐shJAG1 mediated knockdown of Jagged1 but got improved by Ad‐miR‐21 sponge mediated loss of miR‐21. These data indicate that miR‐21 mediates TGF‐β1 induced CMT and myocardial fibrosis by targeting Jagged1.

Up to now, a series of miRNAs have been reported to target Jagged1, but most of these identified miRNAs function to inhibit Notch signalling mediated tumorigenesis. For example, miR‐26a directly targeted Jagged1 and inhibited stem cell‐like phenotype and tumour growth of osteosarcoma.^20^ miR‐186 down‐regulated Jagged1 expression by directly targeting its 3′‐UTR and inhibited multiple myeloma.^21^ More recently, miR‐34a was reported to bind to 3′‐UTR of Jagged1 and attenuated the migration and invasion of colon cancer cells.^22^ However, the targeting of Jagged1 by miRNAs in the regulation of myocardial fibrosis has not been reported. Therefore, our study is the first to demonstrate that miR‐21 regulates myocardial fibrosis by targeting Notch/Jagged1 signalling pathway. Considering that up‐regulation of Jagged1 is indicative of the activation of Notch signalling, our results help explain how TGF‐β1 signalling antagonizes Notch signalling to promote myocardial fibrosis. However, further studies are needed to determine whether previously reported miRNAs targeting Jagged1 also play a role in the regulation of myocardial fibrosis. In addition, it remains unclear whether and how Notch signalling could regulate TGF‐β1/miR‐21 pathway.

Notably, recent studies reveal additional mechanisms underlying the role of miR‐21 in cardiac fibrosis. Cao et al^23^ reported that tumour suppressor cell adhesion molecule 1 (CADM1) is the potential target of miR‐21 and miR‐21 enhances cardiac fibrotic remodelling and fibroblast proliferation via targeting CADM1. Yuan et al^12^ found that miR‐21 promoted cardiac fibrosis after MI via targeting Smad7. In addition, plasma miR‐21 level was significantly higher in patients with acute MI compared with control, suggesting that plasma miR‐21 may be a novel biomarker for acute MI.^24^ Furthermore, gene therapy of cardiovascular disease via the targeting of miR‐21 has been recently tried.^25^ Thus miR‐21 has significant implication for the development of novel therapy strategy for cardiovascular diseases.

In summary, this study for the first time reveals that miR‐21 mediates TGF‐β1 induced CMT and myocardial fibrosis by targeting Jagged1. miR‐21 and Jagged1 are potential therapeutic targets for myocardial fibrosis.

## CONFLICT OF INTEREST

None.
